# Tibial nerve stimulation to inhibit the micturition reflex by an implantable wireless driver microstimulator in cats: Notice of Retraction

**DOI:** 10.1097/01.md.0000510819.12615.2b

**Published:** 2016-11-28

The authors of “Tibial nerve stimulation to inhibit the micturition reflex by an implantable wireless driver microstimulator in cats”^[1]^ have requested that the article be retracted. The authors recently performed further studies about tibial nerve stimulation and found that some results were not in accordance with those data published in the paper.
